# Genome-edited human stem cell-derived beta cells: a powerful tool for drilling down on type 2 diabetes GWAS biology

**DOI:** 10.12688/f1000research.8682.1

**Published:** 2016-07-15

**Authors:** Nicola L. Beer, Anna L. Gloyn

**Affiliations:** 1 Oxford Centre for Diabetes Endocrinology and Metabolism, Churchill Hospital, Oxford, UK; 2 Wellcome Trust Centre for Human Genetics, Oxford, UK; 3 Oxford NIHR Biomedical Research Centre, Churchill Hospital, Oxford, UK

**Keywords:** human endocrine pancreas derivation, Induced pluripotent stem cells, CRISPR-Cas9, maturity-onset diabetes of the young, Wolfram syndrome

## Abstract

Type 2 diabetes (T2D) is a disease of pandemic proportions, one defined by a complex aetiological mix of genetic, epigenetic, environmental, and lifestyle risk factors. Whilst the last decade of T2D genetic research has identified more than 100 loci showing strong statistical association with disease susceptibility, our inability to capitalise upon these signals reflects, in part, a lack of appropriate human cell models for study. This review discusses the impact of two complementary, state-of-the-art technologies on T2D genetic research: the generation of stem cell-derived, endocrine pancreas-lineage cells and the editing of their genomes. Such models facilitate investigation of diabetes-associated genomic perturbations in a physiologically representative cell context and allow the role of both developmental and adult islet dysfunction in T2D pathogenesis to be investigated. Accordingly, we interrogate the role that patient-derived induced pluripotent stem cell models are playing in understanding cellular dysfunction in monogenic diabetes, and how site-specific nucleases such as the clustered regularly interspaced short palindromic repeats (CRISPR)-Cas9 system are helping to confirm genes crucial to human endocrine pancreas development. We also highlight the novel biology gleaned in the absence of patient lines, including an ability to model the whole phenotypic spectrum of diabetes phenotypes occurring both *in utero* and in adult cells, interrogating the non-coding ‘islet regulome’ for disease-causing perturbations, and understanding the role of other islet cell types in aberrant glycaemia. This article aims to reinforce the importance of investigating T2D signals in cell models reflecting appropriate species, genomic context, developmental time point, and tissue type.

## Introduction

### Main question or problem

Type 2 diabetes (T2D) is a global health burden. Given that more than 415 million individuals are currently affected and that the incidence is predicted to rise faster than the adult population growth rate ^1^, it could be argued that our current preventative and therapeutic strategies against this disorder are inadequate.

Understanding T2D pathophysiology is inherently difficult because of its complex aetiology; an individual’s disease risk is based on a combination of genetic, epigenetic, environmental, and lifestyle risk factors ^2, 3^. However, the last decade or so has seen a transformation in our understanding of the genetic basis of this disease; through large-scale international collaborations and DNA samples from hundreds of thousands of individuals, common and rare variant association studies have identified more than 100 genomic loci influencing T2D susceptibility ^4, 5^. Also, for T2D, and unlike many other complex genetic disorders, we have a good handle on the tissue driving pathogenesis; despite perturbations to both insulin secretion and sensitivity, multiple studies place pancreatic islet dysfunction at centre stage in terms of disease susceptibility and progression ^6– 8^.

Despite this wealth of information, our ability to go from genetic signal to mechanism (and even therapeutic target) has progressed at a pace far slower than that of the initial discoveries of these disease susceptibility loci. Why?

### Specifics about the questions or problem

Multiple factors underlie the difficulties in biological interpretation of genome-wide association study data. Firstly, we need to know which transcript(s) are driving the phenotypic signal. This has formed a huge stumbling block for researchers as (i) extensive regions of linkage-disequilibrium mean that most associated loci harbour many genes and transcripts, (ii) many signals lie within poorly annotated, non-coding regions of the genome (although efforts to map the ‘islet regulome’ are beginning to bear fruit ^9– 12^), and (iii) the modest effect sizes of disease-associated variants make functional interrogation of risk versus non-risk alleles problematic (odds ratios are usually between 1.1 and 1.4 ^4, 5^).

Secondly, far and away one of the biggest challenges has been the lack of appropriate human islet cell models for study. Until very recently, this was limited to animal models and rodent insulinoma cell lines, which present numerous challenges; there are multiple instances in which human diabetic phenotypes are not recapitulated in the analogous murine model of gene haploinsufficiency ^13– 24^, and differences in islet architecture, ion channel composition, nutrient sensitivity, and other physiological parameters ^25– 31^ limit the functional inferences that can be made from rodent-derived data. Human islet isolation programmes and the subsequent availability of this tissue for research purposes have gone some way to alleviate this bottleneck, as has the recent generation of human beta-cell lines from pancreas explants ^32, 33^, although these latter cells are only just beginning to be characterised ^34^.

Thirdly, despite increasing access to human islets and cell lines, many technical constraints remain: (i) human islets are heterogeneous in terms of donor genotype and function/viability after surgical extraction, (ii) the restriction of islet isolation programmes to adult donors limits study to mature cells, (iii) human beta-cell lines represent only a single islet cell type, and (iv) low recombination rates and an inability to expand single clones make genomic manipulation via site-specific nucleases challenging.

## What is to come in the rest of the review

This article will focus on one of the most exciting emerging fields in diabetes research at present: human endocrine pancreas derivation in a dish. The utilisation of state-of-the-art *in vitro* differentiation techniques to turn human pluripotent stem cells into those of the islet lineage ^35– 41^ allows researchers to sequentially generate definitive endoderm cells (expressing *SOX17* and *FOXA2*) through to pancreatic progenitors ( *PDX1*- and *NKX6.1*-positive), all the way to cells expressing insulin, glucagon, and islet transcription factors regulating mature cell function ( *MAFA*).

**Figure 1.**
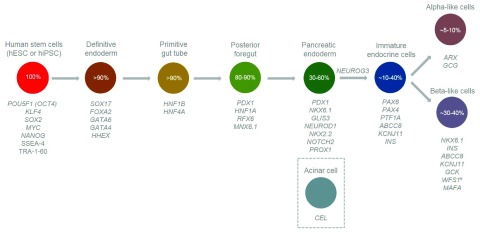
Expression time points for genes important to endocrine pancreas development and diabetes pathology. Circles represent discrete developmental stages, with derivation efficiency estimates also shown ^59^. Genes discussed in this article are listed according to the developmental stage at which they are first expressed and any subsequent stages where they perform important biological functions or are crucial for cell identity ( ^#^except *WFS1* which is expressed at all stages; however, the diabetes observed in patients with Wolfram syndrome is believed to result from selective beta-cell loss via apoptosis ^76^). *CEL* is expressed in acinar cells, which differentiate from multipotent pancreatic progenitor cells and subsequently exocrine progenitor cells (not depicted in the figure). hESC, human embryonic stem cell; hiPSC, human induced pluripotent stem cell.

This model system has broad application in many areas of islet biology and diabetes research. Firstly, it can be used as a platform for drug discovery efforts aimed at increasing functional beta-cell mass, and importantly, one which is without many of the ethical, legal, and practical considerations surrounding the routine use of human tissue (both foetal and adult). Induced pluripotent stem cells (iPSCs) specifically bypass the need for embryonic tissue as they can be generated by reprogramming any somatic cell ^42, 43^. Secondly, the ability to further mature these cells *in vivo*, and to phenotypically correct diabetes in immunocompromised mice ^38– 40, 44– 49^, also shows the translational potential of such cells, with analogous clinical trials beginning to take place in humans ^50^. Both of these areas have been reviewed extensively elsewhere ^51– 56^. Instead, the rest of this article will focus on the potential of stem cell-derived islet-lineage cells in disease modelling, in particular how they can be manipulated with genome editing tools such as CRISPR-Cas9 ^57, 58^, so as to accurately recapitulate the genomic, developmental, and mature cell perturbations underlying T2D pathogenesis ^59^ ( Figure 1).

### Diabetes modelling using patient-derived cells

Recent methodological advances in endocrine pancreas differentiation have promoted formation of mono-hormonal cells with function similar to (but not quite yet the same as) that of human islets ^38– 41^. However, variation in line-to-line differentiation efficiencies ^60– 62^ coupled with an inability to make fully mature cells ^63^ has so far limited disease modelling to monogenic diabetes caused by highly penetrant, large-effect mutations.

One of the first proof-of-principle studies ^64^ generated iPSC lines from individuals with maturity-onset diabetes of the young (MODY) by using a polycistronic lentiviral vector overexpressing the so-called ‘Yamanaka factors’ ( *POU5F1* [OCT4], *KLF4*, *SOX2*, and *MYC*), these needed for somatic cell reprogramming to pluripotency ^42^. This included lines from patients with mutations in endocrine pancreas developmental transcription factors ( *HNF1B*, *HNF4A*, and *HNF1A*), as well as those with perturbed enzymes governing glucose-stimulated insulin secretion (GSIS) in mature cells ( *GCK*), and even exocrine pancreas function ( *CEL*). Regardless of mutated gene, all lines were shown to fulfil basic iPSC quality control: expression of pluripotency genes via fluorescence-activated cell sorting (OCT4, SOX2, NANOG, SSEA-4, and TRA-1-60), spontaneous teratoma formation upon transplant into immunocompromised mice (cells capable of generating all three germ layers), and a diploid ‘stable’ karyotype ^64^. Another study aimed at generating iPSCs from patients with HNF1A-MODY ^65^ again produced cells passing basic pluripotency QC, and which were able to differentiate from embryoid bodies, into those expressing insulin and glucagon. Of note here is that these hormones were not present at levels comparable to those seen in other studies ^39, 40^, perhaps reflecting the quite different *in vitro* differentiation strategies employed. Likewise, the inability of these cells to form teratomas spontaneously *in vivo* suggests that reprogramming to full pluripotency may not have been achieved.

Other diabetes iPSC models have focussed on characterising cellular dysfunction apparent within mature islets, making endocrine pancreas differentiation essential for phenotyping patient-derived cells. Individuals with heterozygous *GCK* mutations have a mild phenotype whereby fasting plasma glucose levels are marginally elevated (6 to 8 mmol/L) because of a higher threshold for GSIS, which is governed by altered beta-cell glucose uptake and glycolytic flux ^66, 67^. Directed differentiation of iPSCs from patients with GCK-MODY down the islet lineage occurred with an efficiency comparable to that of control cells, with the only observable defects mirroring patient phenotype (elevated GSIS set-point), thus validating this as a physiologically representative model for studying monogenic *GCK* mutations ^68^.

iPSC models have also been generated for syndromic diabetes disorders, such as Wolfram syndrome. This disorder is caused by mutations in *WFS1* ^69^, with patients suffering from multi-organ dysfunction, including diabetes, optic atrophy, and neurodevelopmental defects ^70^. Such a broad phenotype reflects the multi-tissue expression of *WFS1*, with the encoded Wolframin protein performing vital roles in endoplasmic reticulum (ER) Ca ^2+^ homeostasis ^71– 73^ as well as alleviating ER stress in cells with high translational load ^74^, such as those with secretory function ^75^. This is thought to explain the childhood-onset diabetes in these individuals, with post-mortem study of Wolfram syndrome pancreases suggesting selective beta-cell loss via apoptosis ^76^. Directed differentiation of iPSCs from patients with Wolfram syndrome down the islet lineage showed that these cells had elevated levels of chemically induced ER stress, which resulted in translational stasis and decreased insulin processing and content. Likewise, *in vivo* maturation of patient cells showed that grafts declined in function much more rapidly than control cells, perhaps reflecting enhanced apoptosis ^77^.

### The need for phenotypic correction of patient stem cells

Importantly, the cellular dysfunction observed in both diabetes iPSC-derived models was corrected via genetic (zinc finger nuclease ^68^) or chemical (4-phenylbutyric acid ^77^) means. This phenotypic correction is fundamental in assigning causality to the studied mutation of interest, particularly as large-scale sequencing studies are continuing to identify previously reported ‘disease-causing’ mutations in unaffected individuals within the general population, leading to continued revision and reduction of penetrance estimates ^78^. Likewise, comparing patient lines to isogenic controls removes any differentiation efficiency or phenotypic effects driven by factors extrinsic to the particular mutation of interest, including reprogramming efficiency and epigenetic or sequence variation (or both) in the donor genome ^60– 62^.

A methodological advance which has revolutionised the ease at which we can generate isogenic control lines is the expansion of site-directed nuclease, so-called ‘genome editing’ technologies, from zinc finger nucleases ^79, 80^ to TALENs (transcription activator-like effector nucleases) ^81– 85^ and more recently to CRISPR-Cas9 ^57, 58, 86– 100^. The most popular of these editing methods, CRISPR-Cas9, exploits a bacterial innate immune system response to pathogens, whereby the Cas9 endonuclease is targeted to invading phage DNA by a sequence-specific guide RNA molecule ^101^. In recent years, manipulation of this system so that it can target eukaryotic (specifically mammalian) genomes has allowed its full translational potential to be realised ^57, 58, 102^. The ability to target more or less any sequence in the human genome for gene knockout via non-homologous end-joining ^103^, nucleotide-level manipulation via homology-directed repair ^104, 105^, or larger recombination events to generate reporter lines ^90, 106^ or even bring into close proximity mediators of gene expression (such as activators or repressors tethered to modified Cas9 protein) ^86– 88, 92, 93, 107^ means that every type of genetic perturbation is theoretically possible. Use of this technology has also extended into simultaneous targeting of multiple genes ^57, 87, 88, 98– 100^ as well as inducible ^89^ and epigenome-modifying ^91, 108^ systems.

Accordingly, CRISPR-Cas9 and other site-specific nucleases are a very attractive tool for the generation or correction (or both) of diabetes-relevant mutations in human stem cell-derived models, stem cells being particularly amenable to this technology because of their clonal nature and highly recombinogenic genome. Both gene knockout via Cas9-induced indels ^109^ and doxycycline-inducible gain-of-function transgenes (targeted to the *AAVS1* safe harbour locus using TALENs ^110^) have been used to definitively establish the role of *NEUROG3* in human pancreas development. Whilst *Neurog3* is essential for murine pancreas development and derivation of all islet cell types ^111– 113^, individuals with homozygous *NEUROG3* mutations retain some islet function ^114– 116^. Complete gene knockout showed that *NEUROG3 ^−/−^* cells could not mature past pancreatic progenitors into endocrine pancreas; however, with graded perturbation to gene dosage via small hairpin RNA (shRNA), as little as 10% residual *NEUROG3* activity still led to some islet hormone-positive cells ^109^. These data are directionally consistent with analogous experiments whereby inducible *NEUROG3* overexpression in human embryonic stem cell (hESC)-derived pancreatic progenitors leads to increased numbers of endocrine pancreas-like cells expressing *INS*, *NKX2.2*, *NEUROD1*, and other relevant islet transcription factors ^110^. Drastically reduced *NEUROG3* levels are therefore sufficient for the development of human islets, an effect not recapitulated in mice.

Although many reports have begun to emerge of mutation introduction or correction via homology-directed repair in both control and patient-derived cell lines, these remain as yet unpublished, perhaps reflecting the low efficiency of this technique and repeated cleavage of repaired sites ^117^, alongside the additional scrutiny of these experimental techniques in terms of off-target effects ^118, 119^.

### Interrogating diabetes pathology in the absence of patient-derived lines

Patient-derived iPSCs facilitate study of the precise mutational mechanisms underlying an individual’s diabetes risk and progression; however, their use so far has been limited to monogenic disease. Although we may not yet have phenotypic resolution to assay dysfunction underlying more complex disease, the ability to generate cellular models of islet development opens up a whole new avenue of investigation for T2D pathogenesis ^59^.

### T2D pathology may result from dysfunction in both foetal and adult islets

We know from studying monogenic diabetes and pancreatic agenesis that there is substantial overlap between the genes causing these phenotypically severe Mendelian disorders and those harbouring more common and incompletely penetrant variants predisposing to T2D risk ^4, 120^. It follows that within these cellular pathways, the extent of perturbation dictates when diabetes presents: either *in utero*/early life if severe or much later as T2D if more subtle.

At the extreme end of this scale is pancreas hypoplasia or even lack of a pancreas completely (agenesis). Haploinsufficiency for *GATA6* is the most common cause of pancreatic agenesis in humans ^14^. Individuals with this haploinsufficiency may also experience cardiac or gastrointestinal abnormalities, reflecting the role of *GATA6* in organogenesis for multiple tissues. As phenotypic presentation of *GATA6* mutation carriers varies (some individuals experience dysfunction in only a subset of these tissues), a potential redundant role for the related transcription factor *GATA4* has been proposed in humans. This hypothesis is well established in mouse development ^121, 122^ but continues to be the subject of debate in humans, despite the identification of individuals with neonatal diabetes (one with pancreatic agenesis) resulting from heterozygous *GATA4* mutations ^20^.

Biallelic inactivation of *RFX6*, a key transcription factor in gut- and pancreatic-endoderm specification, causes both neonatal ^123– 125^ and childhood-onset diabetes ^126^, with phenotype severity correlating with loss of *RFX6* gene dosage, and subsequently islet cell development/hypoplasia ^126^. An elegant CRISPR-Cas9 hESC knockout study showed that loss of *RFX6* alters or delays pancreatic progenitor formation through perturbed *PDX1* induction ^110^, thus implicating RFX6 in the regulation of both foetal and adult islet cell function (in which it helps maintain mature beta-cell identity ^127, 128^). Heterozygous mutations in *HNF1B* ^129^, a gene switched on within cells in the primitive gut tube where it is responsible for regional gut specification and branching morphogenesis as well as later cell fate decisions in multipotent pancreatic progenitors ^130^, cause MODY ^131, 132^, pancreatic hypoplasia/agenesis ^133– 136^, and renal abnormalities ^137– 139^.

*GATA6* and *HNF1B* map to genomic loci implicated in later-onset diabetes ^4, 120^; therefore, understanding their role in foetal and adult human islets is crucial for investigating T2D pathogenesis. Because mice haploinsufficient for *Gata6*, *Gata4*, and *Hnf1b* do not have diabetes ^15– 17^ and with homozygous knockouts causing embryonic lethality ^140– 142^, dual developmental and adult characterisation would not be possible without human cell models representative of both time points.

### Stem cells can be used to model the whole spectrum of diabetes phenotypes

The severity of a diabetes phenotype may be influenced, in part, by the temporal expression pattern of a mutated gene. For example, one of the downstream targets of HNF1B is GLIS3, a zinc finger transcription factor involved in regulating the transient spike in *NEUROG3* expression important for endocrine fate commitment ^130^. Although *GLIS3* mutations have been shown to cause neonatal diabetes and T2D in humans, these same individuals do not experience pancreatic agenesis ^143^, and this fits with the later expression of *GLIS3* (versus *HNF1B*) in the foetal pancreas. This suggests that these individuals are able to make some endocrine pancreas tissue and that disease pathology results from insufficient insulin secretion from a reduced functional beta-cell mass potentially both *in utero* and in adult life. Analogous observations have been made for individuals with mutations in the foetal pancreatic transcription factors *PAX6* ^144^, *NEUROD1* ^145^, *NKX2.2* ^146^, and *MNX1* ^146, 147^.

In a similar vein, heterozygous mutations in other genes important for islet progenitor function can cause the milder phenotype of MODY; this is characterised by onset of non-insulin-dependent diabetes before 25 years of age ^148^. Mutations in HNF family members *HNF4A* and *HNF1A* are the most common cause of MODY in Europeans ^149– 153^, and these genes also map to genomic regions associated with T2D risk ^4, 120^. Whilst both disorders could result from defective insulin secretion from mature islets (the two transcription factors regulate genes governing GSIS ^24^), they also perform distinctive roles in the foetal pancreas, as dictated by discrete spatiotemporal expression patterns for each of the multiple *HNF4A* and *HNF1A* transcript isoforms ^154– 156^. Studying both HNFs in foetal versus adult tissue has also shown big differences in post-translational regulation; in adult islets these two HNF transcription factors regulate expression of each other and themselves ^157^ whereas only *HNF4A* mutations have been shown to cause the more severe phenotype of neonatal diabetes, suggesting that this gene has a more dominant role in foetal pancreas development ^155^. The association of *HNF4A* variants with macrosomia and hypoglycaemia in neonates ^158^ also suggests that perturbations to this gene transiently increase foetal insulin secretion, a phenomenon not observable if studying (i) adult islets alone (as *HNF4A* mutations cause the opposite phenotype of beta-cell dysfunction and hyperglycaemia ^153^) or (ii) rodent pancreas ( *Hnf4a ^+/−^* and *Hnf1a ^+/−^* mice are phenotypically normal ^13, 18, 24^). Accordingly, understanding the temporal relationship between *HNF4A* gene dosage and insulin secretion is fundamental to managing pregnancy as well as neonatal and young-onset diabetes and T2D.

Irrespective of a previous implication in Mendelian diabetes, knowing the developmental expression pattern of genes mapping to T2D-associated regions of the genome can also help refine likely effector transcripts at these loci, particularly considering the well-established role of islet dysfunction in the progression of this disease ^6– 8^. *HHEX*, *NOTCH2*, and *PROX1* map to T2D loci containing multiple putative effector transcripts and potentially causal variants ^4, 120^. Although none of these genes harbour mutations implicated in monogenic diabetes, strong candidacy for their role as effector transcript comes from their importance in endocrine pancreas development: *HHEX* regulates ventral pancreas organogenesis ^159^, *NOTCH2* is involved in fate decisions of pancreatic progenitors ^160^, and *PROX1* marks pancreatic progenitor cells in the endoderm (later becoming specific to *NEUROG3*-positive cells) ^161^. Thus, using human models of endocrine pancreas differentiation to understand how subtle perturbations to these genes during development may impact upon risk of diabetes in later life is fundamental to the functional characterisation, and consequent assignment of variant/transcript causality, at these T2D-associated genomic loci ^59^.

This same principle can be applied to disentangling disease-associated genetic perturbations mapping to non-coding regions of the genome. As many islet enhancers are tissue-specific ^162^, and with studies in stem cell-derived endocrine pancreas-lineage cells also showing these and other regulatory marks to be developmental stage specific too ^163^, it follows that characterisation of non-coding regions harbouring disease-associated genetic variations is possible only if developmental pancreas cell models are employed. A good example of this approach comes from a recent study of multiple consanguineous families with recessive pancreatic agenesis of unknown aetiology ^164^. All affected individuals were absent of coding mutations in previously established pancreatic agenesis genes ( *GATA6* ^14^, *PTF1A* ^165^, and *PDX1* ^166, 167^) and accordingly were subjected to whole genome sequencing. Homozygosity mapping showed that no biallelic coding changes co-segregated with disease. Extended analysis into non-coding regions of the genome showed that multiple affected individuals harboured biallelic mutations in a 400-base pair sequence about 25 kB downstream of *PTF1A*, a transcription factor mediating early pancreas specification from the foregut ^168^. ChIP-seq in hESC-derived pancreatic progenitors showed that this region overlapped binding sites for the foetal pancreas transcription factors *FOXA2* and *PDX1* as well as an H3K4me1 active enhancer site. Enhancer activity was shown to be tissue- and developmental stage-specific and was abolished upon introduction of the agenesis mutations ^164^. As *PTF1A* maps to a locus associated with T2D ^4, 120^, it follows that similar developmental enhancers may also be important in adult-onset disease.

### The usefulness of a model capable of recapitulating all islet cell types

Although as diabetes researchers we can put a large emphasis on understanding insulin secretory defects, aberrant glycaemia can also result from dysfunction in other islet cell types.

Because differentiated stem cells make cells positive for all islet hormones ^39, 40^, one can use the same systems to study aberrant glycaemia resulting from perturbations in non-beta cell types. Diffuse congenital hyperinsulinism in infancy (CHI) is characterised by insulin over-secretion despite hypoglycaemia ^169^. Mutations in the ATP-sensitive islet potassium channel subunit genes *ABCC8* and *KCNJ11* are the most common cause of CHI; the unregulated closure of this channel is thought to result in sustained insulin release ^170, 171^. However, study of pancreas tissue from 10 individuals with *KCNJ11*-mediated CHI showed that functional beta-cell mass was maintained as constant since, despite increased proliferation, a concomitant elevation in cell type-specific apoptosis was also observed ^172^. Intriguingly, and consistent with the disorganised islet architecture observed in *Kcnj11* knockout mice ^21^, the human CHI islets had downregulated *PAX4* and *ARX* levels (the latter transcription factor specific to alpha cells ^173^) as well as elevated *NKX2.2* expression (particularly in delta cells, 10% of which also demonstrated nucleomegaly) ^172, 174, 175^. Consistent with the use of somatostatin analogues in the treatment of some CHI cases ^169^, these data suggest that alteration of multiple endocrine pancreas cell lineages (not just beta cells) is driving phenotype ^172^. Despite disorganised islets, *Kcnj11* and *Abcc8* knockout mice do not exactly recapitulate the phenotype of human CHI ^21, 22^, making the further investigation of this disorder in stem cell-derived endocrine pancreas models attractive.

## Summary

This review highlights the need for human, physiologically relevant cell models which accurately recapitulate both foetal and adult islet function for interrogation of diabetes pathogenesis. Although a lot of our knowledge regarding pancreas development has come from studying the mouse, there are many cases in which murine models fall phenotypically short and so translating genetic signals into disease mechanisms is limited. The huge advances that have been made in differentiating human stem cells (both embryonic and induced pluripotent) into all cell types of the developing endocrine pancreas have transformed how we are able to characterise disease-causing and -associated genetic perturbations. However, although we are now able to make endocrine pancreas-like cells with some islet function, it is important to temper expectations and remember that we are still some way from making the perfect beta cell. Although the most recent studies from leading labs report glucose-responsive insulin secretion and Ca ^2+^ channel activity ^39, 40^, this function does not fully recapitulate that of human islets. Accordingly, we as a field must make an effort to standardise phenotyping assays and subject them to the same scrutiny as that used to interrogate primary tissue. Efforts to deposit functional ^176^ and omics-level ^59^ data for both primary tissue and stem cell-derived endocrine pancreas-like cells are helping researchers generating their own pancreas-in-a-dish to compare, contrast, and truly evaluate their model systems. Once this methodological standardisation is achieved, we can collectively increase the complexity of our routine phenotyping of parameters such as hormone secretion and ion currents and move towards physiologically relevant doses of mixed nutrient stimuli, amongst other assays.

Regardless of these current functional bottlenecks, coupling stem cell-derived endocrine pancreas-like cells with the excitement of genome editing technologies places diabetes researchers in an extremely powerful position of novel biology discovery and genetic signal validation. Armed with these new experimental tools, one can start probing more complex forms of the disease such as T2D ^59^ and, with a pluripotent cell type, model the complex multi-organ dysfunction occurring in cells derived from the same patient. The dream of a true ‘personalised medicine’ approach to diabetes is in our midst.

## References

[1] International Diabetes Federation: IDF Diabetes Atlas - 7th Edition.2015 Reference Source 35914061

[2] Almgren P Lehtovirta M Isomaa B: Heritability and familiality of type 2 diabetes and related quantitative traits in the Botnia Study. *Diabetologia.* 2011;54(11):2811–9. 10.1007/s00125-011-2267-5 21826484

[3] Hu FB: Globalization of diabetes: the role of diet, lifestyle, and genes. *Diabetes Care.* 2011;34(6):1249–57. 10.2337/dc11-0442 21617109 PMC3114340

[4] Morris AP Voight BF Teslovich TM: Large-scale association analysis provides insights into the genetic architecture and pathophysiology of type 2 diabetes. *Nat Genet.* 2012;44(9):981–90. 10.1038/ng.2383 22885922 PMC3442244

[5] DIAbetes Genetics Replication And Meta-analysis (DIAGRAM) Consortium, Asian Genetic Epidemiology Network Type 2 Diabetes (AGEN-T2D) Consortium, South Asian Type 2 Diabetes (SAT2D) Consortium, : Genome-wide trans-ancestry meta-analysis provides insight into the genetic architecture of type 2 diabetes susceptibility. *Nat Genet.* 2014;46(3):234–44. 10.1038/ng.2897 24509480 PMC3969612

[6] Dimas AS Lagou V Barker A: Impact of type 2 diabetes susceptibility variants on quantitative glycemic traits reveals mechanistic heterogeneity. *Diabetes.* 2014;63(6):2158–71. 10.2337/db13-0949 24296717 PMC4030103

[7] Ingelsson E Langenberg C Hivert MF: Detailed physiologic characterization reveals diverse mechanisms for novel genetic Loci regulating glucose and insulin metabolism in humans. *Diabetes.* 2010;59(5):1266–75. 10.2337/db09-1568 20185807 PMC2857908

[8] Voight BF Scott LJ Steinthorsdottir V: Twelve type 2 diabetes susceptibility loci identified through large-scale association analysis. *Nat Genet.* 2010;42(7):579–89. 10.1038/ng.609 20581827 PMC3080658

[9] Fadista J Vikman P Laakso EO: Global genomic and transcriptomic analysis of human pancreatic islets reveals novel genes influencing glucose metabolism. *Proc Natl Acad Sci U S A.* 2014;111(38):13924–9. 10.1073/pnas.1402665111 25201977 PMC4183326

[10] Gaulton KJ Ferreira T Lee Y: Genetic fine mapping and genomic annotation defines causal mechanisms at type 2 diabetes susceptibility loci. *Nat Genet.* 2015;47(12):1415–25. 10.1038/ng.3437 26551672 PMC4666734

[11] Parker SC Stitzel ML Taylor DL: Chromatin stretch enhancer states drive cell-specific gene regulation and harbor human disease risk variants. *Proc Natl Acad Sci U S A.* 2013;110(44):17921–6. 10.1073/pnas.1317023110 24127591 PMC3816444

[12] van de Bunt M Manning Fox JE Dai X: Transcript Expression Data from Human Islets Links Regulatory Signals from Genome-Wide Association Studies for Type 2 Diabetes and Glycemic Traits to Their Downstream Effectors. *PLoS Genet.* 2015;11(12):e1005694. 10.1371/journal.pgen.1005694 26624892 PMC4666611

[13] Dukes ID Sreenan S Roe MW: Defective pancreatic beta-cell glycolytic signaling in hepatocyte nuclear factor-1alpha-deficient mice. *J Biol Chem.* 1998;273(38):24457–64. 10.1074/jbc.273.38.24457 9733737

[14] Lango Allen H Flanagan SE Shaw-Smith C: GATA6 haploinsufficiency causes pancreatic agenesis in humans. *Nat Genet.* 2012;44(1):20–2. 10.1038/ng.1035 22158542 PMC4062962

[15] Kuo CT Morrisey EE Anandappa R: GATA4 transcription factor is required for ventral morphogenesis and heart tube formation. *Genes Dev.* 1997;11(8):1048–60. 10.1101/gad.11.8.1048 9136932

[16] Morrisey EE Tang Z Sigrist K: GATA6 regulates HNF4 and is required for differentiation of visceral endoderm in the mouse embryo. *Genes Dev.* 1998;12(22):3579–90. 10.1101/gad.12.22.3579 9832509 PMC317242

[17] Molkentin JD Lin Q Duncan SA: Requirement of the transcription factor GATA4 for heart tube formation and ventral morphogenesis. *Genes Dev.* 1997;11(8):1061–72. 10.1101/gad.11.8.1061 9136933

[18] Pontoglio M Prié D Cheret C: HNF1alpha controls renal glucose reabsorption in mouse and man. *EMBO Rep.* 2000;1(4):359–65. 10.1093/embo-reports/kvd071 11269503 PMC1083745

[19] Servitja JM Ferrer J: Transcriptional networks controlling pancreatic development and beta cell function. *Diabetologia.* 2004;47(4):597–613. 10.1007/s00125-004-1368-9 15298336

[20] Shaw-Smith C De Franco E Lango Allen H: *GATA4* mutations are a cause of neonatal and childhood-onset diabetes. *Diabetes.* 2014;63(8):2888–94. 10.2337/db14-0061 24696446 PMC6850908

[21] Seino S Iwanaga T Nagashima K: Diverse roles of K _ATP_ channels learned from Kir6.2 genetically engineered mice. *Diabetes.* 2000;49(3):311–8. 10.2337/diabetes.49.3.311 10868950

[22] Seghers V Nakazaki M DeMayo F: *Sur1* knockout mice. A model for K _ATP_ channel-independent regulation of insulin secretion. *J Biol Chem.* 2000;275(13):9270–7. 10.1074/jbc.275.13.9270 10734066

[23] Shiota C Larsson O Shelton KD: Sulfonylurea receptor type 1 knock-out mice have intact feeding-stimulated insulin secretion despite marked impairment in their response to glucose. *J Biol Chem.* 2002;277(40):37176–83. 10.1074/jbc.M206757200 12149271

[24] Stoffel M Duncan SA: The maturity-onset diabetes of the young (MODY1) transcription factor HNF4alpha regulates expression of genes required for glucose transport and metabolism. *Proc Natl Acad Sci USA.* 1997;94(24):13209–14. 10.1073/pnas.94.24.13209 9371825 PMC24288

[25] De Vos A Heimberg H Quartier E: Human and rat beta cells differ in glucose transporter but not in glucokinase gene expression. *J Clin Invest.* 1995;96(5):2489–95. 10.1172/JCI118308 7593639 PMC185903

[26] Hay CW Docherty K: Comparative analysis of insulin gene promoters: implications for diabetes research. *Diabetes.* 2006;55(12):3201–13. 10.2337/db06-0788 17130462

[27] Fiaschi-Taesch N Bigatel TA Sicari B: Survey of the human pancreatic beta-cell G1/S proteome reveals a potential therapeutic role for cdk-6 and cyclin D1 in enhancing human beta-cell replication and function *in vivo*. *Diabetes.* 2009;58(4):882–93. 10.2337/db08-0631 19136653 PMC2661601

[28] McCulloch LJ van de Bunt M Braun M: GLUT2 ( *SLC2A2*) is not the principal glucose transporter in human pancreatic beta cells: implications for understanding genetic association signals at this locus. *Mol Genet Metab.* 2011;104(4):648–53. 10.1016/j.ymgme.2011.08.026 21920790

[29] McDonald TJ Tu E Brenner S: Canine, human, and rat plasma insulin responses to galanin administration: species response differences. *Am J Physiol.* 1994;266(4 Pt 1):E612–7. 7513957 10.1152/ajpendo.1994.266.4.E612

[30] Peschke E Bähr I Mühlbauer E: Melatonin and pancreatic islets: interrelationships between melatonin, insulin and glucagon. *Int J Mol Sci.* 2013;14(4):6981–7015. 10.3390/ijms14046981 23535335 PMC3645673

[31] Steiner DJ Kim A Miller K: Pancreatic islet plasticity: interspecies comparison of islet architecture and composition. *Islets.* 2010;2(3):135–45. 10.4161/isl.2.3.11815 20657742 PMC2908252

[32] Ravassard P Hazhouz Y Pechberty S: A genetically engineered human pancreatic β cell line exhibiting glucose-inducible insulin secretion. *J Clin Invest.* 2011;121(9):3589–97. 10.1172/JCI58447 21865645 PMC3163974

[33] Scharfmann R Pechberty S Hazhouz Y: Development of a conditionally immortalized human pancreatic β cell line. *J Clin Invest.* 2014;124(5):2087–98. 10.1172/JCI72674 24667639 PMC4001549

[34] Andersson LE Valtat B Bagge A: Characterization of stimulus-secretion coupling in the human pancreatic EndoC-βH1 beta cell line. *PLoS One.* 2015;10(3):e0120879. 10.1371/journal.pone.0120879 25803449 PMC4372368

[35] Bruin JE Erener S Vela J: Characterization of polyhormonal insulin-producing cells derived *in vitro* from human embryonic stem cells. *Stem Cell Res.* 2014;12(1):194–208. 10.1016/j.scr.2013.10.003 24257076

[36] Cho CH Hannan NR Docherty FM: Inhibition of activin/nodal signalling is necessary for pancreatic differentiation of human pluripotent stem cells. *Diabetologia.* 2012;55(12):3284–95. 10.1007/s00125-012-2687-x 23011350 PMC3483105

[37] D'Amour KA Bang AG Eliazer S: Production of pancreatic hormone-expressing endocrine cells from human embryonic stem cells. *Nat Biotechnol.* 2006;24(11):1392–401. 10.1038/nbt1259 17053790

[38] Nostro MC Sarangi F Yang C: Efficient generation of NKX6–1 ^+^ pancreatic progenitors from multiple human pluripotent stem cell lines. *Stem Cell Reports.* 2015;4(4):591–604. 10.1016/j.stemcr.2015.02.017 25843049 PMC4400642

[39] Pagliuca FW Millman, JR Gürtle M: Generation of functional human pancreatic β cells *in vitro.* *Cell.* 2014;159(2):428–39. 10.1016/j.cell.2014.09.040 25303535 PMC4617632

[40] Rezania A Bruin JE Arora P: Reversal of diabetes with insulin-producing cells derived *in vitro* from human pluripotent stem cells. *Nat Biotechnol.* 2014;32(11):1121–33. 10.1038/nbt.3033 25211370

[41] Russ HA Parent AV Ringler JJ: Controlled induction of human pancreatic progenitors produces functional beta-like cells *in vitro*. *EMBO J.* 2015;34(13):1759–72. 10.15252/embj.201591058 25908839 PMC4516429

[42] Takahashi K Tanabe K Ohnuki M: Induction of pluripotent stem cells from adult human fibroblasts by defined factors. *Cell.* 2007;131(5):861–72. 10.1016/j.cell.2007.11.019 18035408

[43] Takahashi K Yamanaka S: Induction of pluripotent stem cells from mouse embryonic and adult fibroblast cultures by defined factors. *Cell.* 2006;126(4):663–76. 10.1016/j.cell.2006.07.024 16904174

[44] Bruin JE Rezania A Xu J: Maturation and function of human embryonic stem cell-derived pancreatic progenitors in macroencapsulation devices following transplant into mice. *Diabetologia.* 2013;56(9):1987–98. 10.1007/s00125-013-2955-4 23771205

[45] Kelly OG Chan MY Martinson LA: Cell-surface markers for the isolation of pancreatic cell types derived from human embryonic stem cells. *Nat Biotechnol.* 2011;29(8):750–6. 10.1038/nbt.1931 21804561

[46] Kroon E Martinson LA Kadoya K: Pancreatic endoderm derived from human embryonic stem cells generates glucose-responsive insulin-secreting cells *in vivo*. *Nat Biotechnol.* 2008;26(4):443–52. 10.1038/nbt1393 18288110

[47] Nostro MC Sarangi F Ogawa S: Stage-specific signaling through TGFβ family members and WNT regulates patterning and pancreatic specification of human pluripotent stem cells. *Development.* 2011;138(5):861–71. 10.1242/dev.055236 21270052 PMC3035090

[48] Rezania A Bruin JE Riedel MJ: Maturation of human embryonic stem cell-derived pancreatic progenitors into functional islets capable of treating pre-existing diabetes in mice. *Diabetes.* 2012;61(8):2016–29. 10.2337/db11-1711 22740171 PMC3402300

[49] Rezania A Bruin JE Xu J: Enrichment of human embryonic stem cell-derived NKX6.1–expressing pancreatic progenitor cells accelerates the maturation of insulin-secreting cells *in vivo*. *Stem Cells.* 2013;31(11):2432–42. 10.1002/stem.1489 23897760

[50] Viacyte: A Safety, Tolerability, and Efficacy Study of VC-01™ Combination Product in Subjects With Type I Diabetes Mellitus. In ClinicalTrials.gov [Internet]. National Library of Medicine (US), Bethesda (MD),2014;2016 Reference Source

[51] Bruin JE Rezania A Kieffer TJ: Replacing and safeguarding pancreatic β cells for diabetes. *Sci Transl Med.* 2015;7(316):316ps23. 10.1126/scitranslmed.aaa9359 26631630

[52] Cogger K Nostro MC: Recent advances in cell replacement therapies for the treatment of type 1 diabetes. *Endocrinology.* 2015;156(1):8–15. 10.1210/en.2014-1691 25386833

[53] Nair G Hebrok M: Islet formation in mice and men: lessons for the generation of functional insulin-producing β-cells from human pluripotent stem cells. *Curr Opin Genet Dev.* 2015;32:171–80. 10.1016/j.gde.2015.03.004 25909383 PMC4523641

[54] Pagliuca FW Melton DA: How to make a functional β-cell. *Development.* 2013;140(12):2472–83. 10.1242/dev.093187 23715541 PMC3666377

[55] Quiskamp N Bruin JE Kieffer TJ: Differentiation of human pluripotent stem cells into β-cells: Potential and challenges. *Best Pract Res Clin Endocrinol Metab.* 2015;29(6):833–47. 10.1016/j.beem.2015.10.011 26696513

[56] Robinton DA Daley GQ: The promise of induced pluripotent stem cells in research and therapy. *Nature.* 2012;481(7381):295–305. 10.1038/nature10761 22258608 PMC3652331

[57] Cong L Ran FA Cox D: Multiplex genome engineering using CRISPR/Cas systems. *Science.* 2013;339(6121):819–23. 10.1126/science.1231143 23287718 PMC3795411

[58] Mali P Yang L Esvelt KM: RNA-guided human genome engineering via Cas9. *Science.* 2013;339(6121):823–6. 10.1126/science.1232033 23287722 PMC3712628

[59] van de Bunt M Lako M Barrett A: Insights into islet development and biology through characterization of a human iPSC-derived endocrine pancreas model. *Islets.* 2016;8(3):83–95. 10.1080/19382014.2016.1182276 27246810 PMC4987020

[60] Kajiwara M Aoi T Okita K: Donor-dependent variations in hepatic differentiation from human-induced pluripotent stem cells. *Proc Natl Acad Sci U S A.* 2012;109(31):12538–43. 10.1073/pnas.1209979109 22802639 PMC3411998

[61] Kyttala A Moraghebi R Valensisi C: Genetic Variability Overrides the Impact of Parental Cell Type and Determines iPSC Differentiation Potential. *Stem Cell Reports.* 2016;6(2):200–12. 10.1016/j.stemcr.2015.12.009 26777058 PMC4750096

[62] Rouhani F Kumasaka N de Brito MC: Genetic background drives transcriptional variation in human induced pluripotent stem cells. *PLoS Genet.* 2014;10(6):e1004432. 10.1371/journal.pgen.1004432 24901476 PMC4046971

[63] Hrvatin S O'Donnell CW Deng F: Differentiated human stem cells resemble fetal, not adult, β cells. *Proc Natl Acad Sci U S A.* 2014;111(8):3038–43. 10.1073/pnas.1400709111 24516164 PMC3939927

[64] Teo AK Windmueller R Johansson BB: Derivation of human induced pluripotent stem cells from patients with maturity onset diabetes of the young. *J Biol Chem.* 2013;288(8):5353–6. 10.1074/jbc.C112.428979 23306198 PMC3581399

[65] Stepniewski J Kachamakova-Trojanowska N Ogrocki D: Induced pluripotent stem cells as a model for diabetes investigation. *Sci Rep.* 2015;5:8597. 10.1038/srep08597 25716801 PMC4341212

[66] Matschinsky FM: Regulation of pancreatic beta-cell glucokinase: from basics to therapeutics. *Diabetes.* 2002;51(Suppl 3):S394–404. 10.2337/diabetes.51.2007.S394 12475782

[67] Stride A Vaxillaire M Tuomi T: The genetic abnormality in the beta cell determines the response to an oral glucose load. *Diabetologia.* 2002;45(3):427–35. 10.1007/s00125-001-0770-9 11914749

[68] Hua H Shang L Martinez H: iPSC-derived β cells model diabetes due to glucokinase deficiency. *J Clin Invest.* 2013;123(7):3146–53. 10.1172/JCI67638 23778137 PMC3696557

[69] Inoue H Tanizawa Y Wasson J: A gene encoding a transmembrane protein is mutated in patients with diabetes mellitus and optic atrophy (Wolfram syndrome). *Nat Genet.* 1998;20(2):143–8. 10.1038/2441 9771706

[70] Barrett TG Bundey SE: Wolfram (DIDMOAD) syndrome. *J Med Genet.* 1997;34(10):838–41. 10.1136/jmg.34.10.838 9350817 PMC1051091

[71] Takeda K Inoue H Tanizawa Y: WFS1 (Wolfram syndrome 1) gene product: predominant subcellular localization to endoplasmic reticulum in cultured cells and neuronal expression in rat brain. *Hum Mol Genet.* 2001;10(5):477–84. 10.1093/hmg/10.5.477 11181571

[72] Takei D Ishihara H Yamaguchi S: WFS1 protein modulates the free Ca ^2+^ concentration in the endoplasmic reticulum. *FEBS Lett.* 2006;580(24):5635–40. 10.1016/j.febslet.2006.09.007 16989814

[73] Yurimoto S Hatano N Tsuchiya M: Identification and characterization of wolframin, the product of the wolfram syndrome gene ( *WFS1*), as a novel calmodulin-binding protein. *Biochemistry.* 2009;48(18):3946–55. 10.1021/bi900260y 19292454

[74] Fonseca SG Ishigaki S Oslowski CM: Wolfram syndrome 1 gene negatively regulates ER stress signaling in rodent and human cells. *J Clin Invest.* 2010;120(3):744–55. 10.1172/JCI39678 20160352 PMC2827948

[75] Fonseca SG Fukuma M Lipson KL: WFS1 is a novel component of the unfolded protein response and maintains homeostasis of the endoplasmic reticulum in pancreatic beta-cells. *J Biol Chem.* 2005;280(47):39609–15. 10.1074/jbc.M507426200 16195229

[76] Karasik A O'Hara C Srikanta S: Genetically programmed selective islet beta-cell loss in diabetic subjects with Wolfram's syndrome. *Diabetes Care.* 1989;12(2):135–8. 10.2337/diacare.12.2.135 2649325

[77] Shang L Hua H Foo K: β-cell dysfunction due to increased ER stress in a stem cell model of Wolfram syndrome. *Diabetes.* 2014;63(3):923–33. 10.2337/db13-0717 24227685 PMC3931392

[78] Flannick J Beer NL Bick AG: Assessing the phenotypic effects in the general population of rare variants in genes for a dominant Mendelian form of diabetes. *Nat Genet.* 2013;45(11):1380–5. 10.1038/ng.2794 24097065 PMC4051627

[79] Porteus MH Carroll D: Gene targeting using zinc finger nucleases. *Nat Biotechnol.* 2005;23(8):967–73. 10.1038/nbt1125 16082368

[80] Urnov FD Rebar EJ Holmes MC: Genome editing with engineered zinc finger nucleases. *Nat Rev Genet.* 2010;11(9):636–46. 10.1038/nrg2842 20717154

[81] Cermak T Doyle EL Christian M: Efficient design and assembly of custom TALEN and other TAL effector-based constructs for DNA targeting. *Nucleic Acids Res.* 2011;39(12):e82. 10.1093/nar/gkr218 21493687 PMC3130291

[82] Hockemeyer D Wang H Kiani S: Genetic engineering of human pluripotent cells using TALE nucleases. *Nat Biotechnol.* 2011;29(8):731–4. 10.1038/nbt.1927 21738127 PMC3152587

[83] Joung JK Sander JD: TALENs: a widely applicable technology for targeted genome editing. *Nat Rev Mol Cell Biol.* 2013;14(1):49–55. 10.1038/nrm3486 23169466 PMC3547402

[84] Miller JC Tan S Qiao G: A TALE nuclease architecture for efficient genome editing. *Nat Biotechnol.* 2011;29(2):143–8. 10.1038/nbt.1755 21179091

[85] Reyon D Tsai SQ Khayter C: FLASH assembly of TALENs for high-throughput genome editing. *Nat Biotechnol.* 2012;30(5):460–5. 10.1038/nbt.2170 22484455 PMC3558947

[86] Balboa D Weltner J Eurola S: Conditionally Stabilized dCas9 Activator for Controlling Gene Expression in Human Cell Reprogramming and Differentiation. *Stem Cell Reports.* 2015;5(3):448–59. 10.1016/j.stemcr.2015.08.001 26352799 PMC4618656

[87] Cheng AW Wang H Yang H: Multiplexed activation of endogenous genes by CRISPR-on, an RNA-guided transcriptional activator system. *Cell Res.* 2013;23(10):1163–71. 10.1038/cr.2013.122 23979020 PMC3790238

[88] Dahlman JE Abudayyeh OO Joung J: Orthogonal gene knockout and activation with a catalytically active Cas9 nuclease. *Nat Biotechnol.* 2015;33(11):1159–61. 10.1038/nbt.3390 26436575 PMC4747789

[89] González F Zhu Z Shi ZD: An iCRISPR platform for rapid, multiplexable, and inducible genome editing in human pluripotent stem cells. *Cell Stem Cell.* 2014;15(2):215–26. 10.1016/j.stem.2014.05.018 24931489 PMC4127112

[90] He X Tan C Wang F: Knock-in of large reporter genes in human cells via CRISPR/Cas9-induced homology-dependent and independent DNA repair. *Nucleic Acids Res.* 2016;44(9):e85. 10.1093/nar/gkw064 26850641 PMC4872082

[91] Hilton IB D'Ippolito AM Vockley CM: Epigenome editing by a CRISPR-Cas9-based acetyltransferase activates genes from promoters and enhancers. *Nat Biotechnol.* 2015;33(5):510–7. 10.1038/nbt.3199 25849900 PMC4430400

[92] Kearns NA Genga RM Enuameh MS: Cas9 effector-mediated regulation of transcription and differentiation in human pluripotent stem cells. *Development.* 2014;141(1):219–23. 10.1242/dev.103341 24346702 PMC3865759

[93] Konermann S Brigham MD Trevino AE: Genome-scale transcriptional activation by an engineered CRISPR-Cas9 complex. *Nature.* 2015;517(7536):583–8. 10.1038/nature14136 25494202 PMC4420636

[94] Mali P Aach J Stranges PB: CAS9 transcriptional activators for target specificity screening and paired nickases for cooperative genome engineering. *Nat Biotechnol.* 2013;31(9):833–8. 10.1038/nbt.2675 23907171 PMC3818127

[95] Pyzocha NK Ran FA Hsu PD: RNA-guided genome editing of mammalian cells. *Methods Mol Biol.* 2014;1114:269–77. 10.1007/978-1-62703-761-7_17 24557909

[96] Ran FA Hsu PD Wright J: Genome engineering using the CRISPR-Cas9 system. *Nat Protoc.* 2013;8(11):2281–308. 10.1038/nprot.2013.143 24157548 PMC3969860

[97] Ran FA Hsu PD Lin CY: Double nicking by RNA-guided CRISPR Cas9 for enhanced genome editing specificity. *Cell.* 2013;154(6):1380–9. 10.1016/j.cell.2013.08.021 23992846 PMC3856256

[98] Sanjana NE Shalem O Zhang F: Improved vectors and genome-wide libraries for CRISPR screening. *Nat Methods.* 2014;11(8):783–4. 10.1038/nmeth.3047 25075903 PMC4486245

[99] Shalem O Sanjana NE Hartenian E: Genome-scale CRISPR-Cas9 knockout screening in human cells. *Science.* 2014;343(616):84–7. 10.1126/science.1247005 24336571 PMC4089965

[100] Shalem O Sanjana NE Zhang F: High-throughput functional genomics using CRISPR-Cas9. *Nat Rev Genet.* 2015;16(5):299–311. 10.1038/nrg3899 25854182 PMC4503232

[101] Deltcheva E Chylinski K Sharma CM: CRISPR RNA maturation by *trans*-encoded small RNA and host factor RNase III. *Nature.* 2011;471(7340):602–7. 10.1038/nature09886 21455174 PMC3070239

[102] Hsu PD Lander ES Zhang F: Development and applications of CRISPR-Cas9 for genome engineering. *Cell.* 2014;157(6):1262–78. 10.1016/j.cell.2014.05.010 24906146 PMC4343198

[103] Bibikova M Golic M Golic KG: Targeted chromosomal cleavage and mutagenesis in Drosophila using zinc-finger nucleases. *Genetics.* 2002;161(3):1169–75. 12136019 10.1093/genetics/161.3.1169 PMC1462166

[104] Liang F Han M Romanienko PJ: Homology-directed repair is a major double-strand break repair pathway in mammalian cells. *Proc Natl Acad Sci U S A.* 1998;95(9):5172–7. 10.1073/pnas.95.9.5172 9560248 PMC20233

[105] San FJ Sung P Klein H: Mechanism of eukaryotic homologous recombination. *Annu Rev Biochem.* 2008;77:229–57. 10.1146/annurev.biochem.77.061306.125255 18275380

[106] Liu H Yang H Zhu D: Systematically labeling developmental stage-specific genes for the study of pancreatic beta-cell differentiation from human embryonic stem cells. *Cell Res.* 2014;24(10):1181–200. 10.1038/cr.2014.118 25190258 PMC4185345

[107] Maeder ML Linder SJ Cascio VM: CRISPR RNA-guided activation of endogenous human genes. *Nat Methods.* 2013;10(10):977–9. 10.1038/nmeth.2598 23892898 PMC3794058

[108] Konermann S Brigham MD Trevino AE: Optical control of mammalian endogenous transcription and epigenetic states. *Nature.* 2013;500(7463):472–6. 10.1038/nature12466 23877069 PMC3856241

[109] McGrath PS Watson CL Ingram C: The Basic Helix-Loop-Helix Transcription Factor NEUROG3 Is Required for Development of the Human Endocrine Pancreas. *Diabetes.* 2015;64(7):2497–505. 10.2337/db14-1412 25650326 PMC4477351

[110] Zhu Z Li QV Lee K: Genome Editing of Lineage Determinants in Human Pluripotent Stem Cells Reveals Mechanisms of Pancreatic Development and Diabetes. *Cell Stem Cell.* 2016;18(6):755–68. 10.1016/j.stem.2016.03.015 27133796 PMC4892994

[111] Gradwohl G Dierich A LeMeur M: *neurogenin3* is required for the development of the four endocrine cell lineages of the pancreas. *Proc Natl Acad Sci U S A.* 2000;97(4):1607–11. 10.1073/pnas.97.4.1607 10677506 PMC26482

[112] Lee JC Smith SB Watada H: Regulation of the pancreatic pro-endocrine gene *neurogenin3*. *Diabetes.* 2001;50(5):928–36. 10.2337/diabetes.50.5.928 11334435

[113] Xu X D'Hoker J Stange G: Beta cells can be generated from endogenous progenitors in injured adult mouse pancreas. *Cell.* 2008;132(2):197–207. 10.1016/j.cell.2007.12.015 18243096

[114] Pinney SE Oliver-Krasinski J Ernst L: Neonatal diabetes and congenital malabsorptive diarrhea attributable to a novel mutation in the human neurogenin-3 gene coding sequence. *J Clin Endocrinol Metab.* 2011;96(7):1960–5. 10.1210/jc.2011-0029 21490072 PMC3135203

[115] Rubio-Cabezas O Jensen JN Hodgson MI: Permanent Neonatal Diabetes and Enteric Anendocrinosis Associated With Biallelic Mutations in *NEUROG3*. *Diabetes.* 2011;60(4):1349–53. 10.2337/db10-1008 21378176 PMC3064109

[116] Wang J Cortina G Wu SV: Mutant neurogenin-3 in congenital malabsorptive diarrhea. *N Engl J Med.* 2006;355(3):270–80. 10.1056/NEJMoa054288 16855267

[117] Isalan M: Zinc-finger nucleases: how to play two good hands. *Nat Methods.* 2012;9(1):32–4. 10.1038/nmeth.1805 22205514

[118] Smith C Gore A Yan W: Whole-genome sequencing analysis reveals high specificity of CRISPR/Cas9 and TALEN-based genome editing in human iPSCs. *Cell Stem Cell.* 2014;15(1):12–3. 10.1016/j.stem.2014.06.011 24996165 PMC4338993

[119] Veres A Gosis BS Ding Q: Low incidence of off-target mutations in individual CRISPR-Cas9 and TALEN targeted human stem cell clones detected by whole-genome sequencing. *Cell Stem Cell.* 2014;15(1):27–30. 10.1016/j.stem.2014.04.020 24996167 PMC4082799

[120] Mahajan A Sim X Ng HJ: Identification and functional characterization of *G6PC2* coding variants influencing glycemic traits define an effector transcript at the *G6PC2-ABCB11* locus. *PLoS Genet.* 2015;11(1):e1004876. 10.1371/journal.pgen.1004876 25625282 PMC4307976

[121] Carrasco M Delgado I Soria B: GATA4 and GATA6 control mouse pancreas organogenesis. *J Clin Invest.* 2012;122(10):3504–15. 10.1172/JCI63240 23006330 PMC3461915

[122] Xuan S Borok MJ Decker KJ: Pancreas-specific deletion of mouse *Gata4* and *Gata6* causes pancreatic agenesis. *J Clin Invest.* 2012;122(10):3516–28. 10.1172/JCI63352 23006325 PMC3461916

[123] Concepcion JP Reh CS Daniels M: Neonatal diabetes, gallbladder agenesis, duodenal atresia, and intestinal malrotation caused by a novel homozygous mutation in *RFX6*. *Pediatr Diabetes.* 2014;15(1):67–72. 10.1111/pedi.12063 23914949 PMC3871990

[124] Smith SB Qu HQ Taleb N: Rfx6 directs islet formation and insulin production in mice and humans. *Nature.* 2010;463(7282):775–80. 10.1038/nature08748 20148032 PMC2896718

[125] Spiegel R Dobbie A Hartman C: Clinical characterization of a newly described neonatal diabetes syndrome caused by *RFX6* mutations. *Am J Med Genet A.* 2011;155A(11):2821–5. 10.1002/ajmg.a.34251 21965172

[126] Sansbury FH Kirel B Caswell R: Biallelic *RFX6* mutations can cause childhood as well as neonatal onset diabetes mellitus. *Eur J Hum Genet.* 2015;23(12):1744–8. 10.1038/ejhg.2015.161 26264437 PMC4795203

[127] Chandra V Albagli-Curiel O Hastoy B: RFX6 regulates insulin secretion by modulating Ca ^2+^ homeostasis in human β cells. *Cell Rep.* 2014;9(6):2206–18. 10.1016/j.celrep.2014.11.010 25497100

[128] Piccand J Strasser P Hodson DJ: Rfx6 maintains the functional identity of adult pancreatic β cells. *Cell Rep.* 2014;9(6):2219–32. 10.1016/j.celrep.2014.11.033 25497096 PMC4542305

[129] Bellanne-Chantelot C Chauveau D Gautier JF: Clinical spectrum associated with hepatocyte nuclear factor-1beta mutations. *Ann Intern Med.* 2004;140(7):510–7. 10.7326/0003-4819-140-7-200404060-00009 15068978

[130] De Vas MG Kopp JL Heliot C: Hnf1b controls pancreas morphogenesis and the generation of Ngn3 ^+^ endocrine progenitors. *Development.* 2015;142(5):871–82. 10.1242/dev.110759 25715395 PMC4352981

[131] Bellanné-Chantelot C Clauin S Chauveau D: Large genomic rearrangements in the hepatocyte nuclear factor-1beta ( *TCF2*) gene are the most frequent cause of maturity-onset diabetes of the young type 5. *Diabetes.* 2005;54(11):3126–32. 10.2337/diabetes.54.11.3126 16249435

[132] Horikawa Y Iwasaki N Hara M: Mutation in hepatocyte nuclear factor-1 beta gene (TCF2) associated with MODY. *Nat Genet.* 1997;17(4):384–5. 10.1038/ng1297-384 9398836

[133] Body-Bechou D Loget P D'Herve D: TCF2/HNF-1beta mutations: 3 cases of fetal severe pancreatic agenesis or hypoplasia and multicystic renal dysplasia. *Prenat Diagn.* 2014;34(1):90–3. 10.1002/pd.4264 24382792

[134] Edghill EL Bingham C Slingerland AS: Hepatocyte nuclear factor-1 beta mutations cause neonatal diabetes and intrauterine growth retardation: support for a critical role of HNF-1beta in human pancreatic development. *Diabet Med.* 2006;23(12):1301–6. 10.1111/j.1464-5491.2006.01999.x 17116179

[135] Haumaitre C Fabre M Cormier S: Severe pancreas hypoplasia and multicystic renal dysplasia in two human fetuses carrying novel *HNF1beta/MODY5* mutations. *Hum Mol Genet.* 2006;15(15):2363–75. 10.1093/hmg/ddl161 16801329

[136] Yorifuji T Kurokawa K Mamada M: Neonatal diabetes mellitus and neonatal polycystic, dysplastic kidneys: Phenotypically discordant recurrence of a mutation in the hepatocyte nuclear factor-1beta gene due to germline mosaicism. *J Clin Endocrinol Metab.* 2004;89(6):2905–8. 10.1210/jc.2003-031828 15181075

[137] Bingham C Bulman MP Ellard S: Mutations in the hepatocyte nuclear factor-1beta gene are associated with familial hypoplastic glomerulocystic kidney disease. *Am J Hum Genet.* 2001;68(1):219–24. 10.1086/316945 11085914 PMC1234916

[138] Carbone I Cotellessa M Barella C: A novel hepatocyte nuclear factor-1beta (MODY-5) gene mutation in an Italian family with renal dysfunctions and early-onset diabetes. *Diabetologia.* 2002;45(1):153–4. 10.1007/s125-002-8258-8 11845237

[139] Nishigori H Yamada S Kohama T: Frameshift mutation, A263fsinsGG, in the hepatocyte nuclear factor-1beta gene associated with diabetes and renal dysfunction. *Diabetes.* 1998;47(8):1354–5. 10.2337/diab.47.8.1354 9703339

[140] Barbacci E Reber M Ott MO: Variant hepatocyte nuclear factor 1 is required for visceral endoderm specification. *Development.* 1999;126(21):4795–805. 10518496 10.1242/dev.126.21.4795

[141] Coffinier C Thepot D Babinet C: Essential role for the homeoprotein vHNF1/HNF1beta in visceral endoderm differentiation. *Development.* 1999;126(21):4785–94. 10518495 10.1242/dev.126.21.4785

[142] Haumaitre C Barbacci E Jenny M: Lack of TCF2/vHNF1 in mice leads to pancreas agenesis. *Proc Natl Acad Sci U S A.* 2005;102(5):1490–5. 10.1073/pnas.0405776102 15668393 PMC547822

[143] Senee V Chelala C Duchatelet S: Mutations in *GLIS3* are responsible for a rare syndrome with neonatal diabetes mellitus and congenital hypothyroidism. *Nat Genet.* 2006;38(6):682–7. 10.1038/ng1802 16715098

[144] Solomon BD Pineda-Alvarez DE Balog JZ: Compound heterozygosity for mutations in *PAX6* in a patient with complex brain anomaly, neonatal diabetes mellitus, and microophthalmia. *Am J Med Genet A.* 2009;149A(11):2543–6. 10.1002/ajmg.a.33081 19876904 PMC2783496

[145] Rubio-Cabezas O Minton JA Kantor I: Homozygous mutations in *NEUROD1* are responsible for a novel syndrome of permanent neonatal diabetes and neurological abnormalities. *Diabetes.* 2010;59(9):2326–31. 10.2337/db10-0011 20573748 PMC2927956

[146] Flanagan SE De Franco E Lango AH: Analysis of transcription factors key for mouse pancreatic development establishes *NKX2-2* and *MNX1* mutations as causes of neonatal diabetes in man. *Cell Metab.* 2014;19(1):146–54. 10.1016/j.cmet.2013.11.021 24411943 PMC3887257

[147] Bonnefond A Vaillant E Philippe J: Transcription factor gene *MNX1* is a novel cause of permanent neonatal diabetes in a consanguineous family. *Diabetes Metab.* 2013;39(3):276–80. 10.1016/j.diabet.2013.02.007 23562494

[148] Tattersall R: Maturity-onset diabetes of the young: a clinical history. *Diabet Med.* 1998;15(1):11–4. 10.1002/(SICI)1096-9136(199801)15:1<11::AID-DIA561>3.0.CO;2-0 9472858

[149] Frayling TM Bulamn MP Ellard S: Mutations in the hepatocyte nuclear factor-1alpha gene are a common cause of maturity-onset diabetes of the young in the U.K. *Diabetes.* 1997;46(4):720–5. 10.2337/diab.46.4.720 9075818

[150] Kropff J Selwood MP McCarthy MI: Prevalence of monogenic diabetes in young adults: a community-based, cross-sectional study in Oxfordshire, UK. *Diabetologia.* 2011;54(5):1261–3. 10.1007/s00125-011-2090-z 21350841

[151] Shields BM Hicks S Shepherd MH: Maturity-onset diabetes of the young (MODY): how many cases are we missing? *Diabetologia.* 2010;53(12):2504–8. 10.1007/s00125-010-1799-4 20499044

[152] Yamagata K Oda N Kaisaki PJ: Mutations in the hepatocyte nuclear factor-1alpha gene in maturity-onset diabetes of the young (MODY3). *Nature.* 1996;384(6608):455–8. 10.1038/384455a0 8945470

[153] Yamagata K Furuta H Oda N: Mutations in the hepatocyte nuclear factor-4alpha gene in maturity-onset diabetes of the young (MODY1). *Nature.* 1996;384(6608):458–60. 10.1038/384458a0 8945471

[154] Harries LW Ellard S Stride A: Isomers of the *TCF1* gene encoding hepatocyte nuclear factor-1 alpha show differential expression in the pancreas and define the relationship between mutation position and clinical phenotype in monogenic diabetes. *Hum Mol Genet.* 2006;15(14):2216–24. 10.1093/hmg/ddl147 16760222

[155] Harries LW Locke JM Shields B: The diabetic phenotype in *HNF4A* mutation carriers is moderated by the expression of *HNF4A* isoforms from the P1 promoter during fetal development. *Diabetes.* 2008;57(6):1745–52. 10.2337/db07-1742 18356407

[156] Harries LW Brown JE Gloyn AL: Species-specific differences in the expression of the *HNF1A*, *HNF1B* and *HNF4A* genes. *PLoS One.* 2009;4(11):e7855. 10.1371/journal.pone.0007855 19924231 PMC2773013

[157] Ferrer J: A genetic switch in pancreatic beta-cells: implications for differentiation and haploinsufficiency. *Diabetes.* 2002;51(8):2355–62. 10.2337/diabetes.51.8.2355 12145145

[158] Pearson ER Boj SF Steele AM: Macrosomia and hyperinsulinaemic hypoglycaemia in patients with heterozygous mutations in the *HNF4A* gene. *PLoS Med.* 2007;4(4):e118. 10.1371/journal.pmed.0040118 17407387 PMC1845156

[159] Bort R Martinez-Barbera JP Beddington RS: *Hex* homeobox gene-dependent tissue positioning is required for organogenesis of the ventral pancreas. *Development.* 2004;131(4):797–806. 10.1242/dev.00965 14736744

[160] Cras-Meneur C Li L Kopan R: Presenilins, Notch dose control the fate of pancreatic endocrine progenitors during a narrow developmental window. *Genes Dev.* 2009;23(17):2088–101. 10.1101/gad.1800209 19723764 PMC2751975

[161] Wang J Kilic G Aydin M: Prox1 activity controls pancreas morphogenesis and participates in the production of "secondary transition" pancreatic endocrine cells. *Dev Biol.* 2005;286(1):182–94. 10.1016/j.ydbio.2005.07.021 16122728

[162] Pasquali L Gaulton KJ Rodriguez-Segui SA: Pancreatic islet enhancer clusters enriched in type 2 diabetes risk-associated variants. *Nat Genet.* 2014;46(2):136–43. 10.1038/ng.2870 24413736 PMC3935450

[163] Wang A Yue F Li Y: Epigenetic priming of enhancers predicts developmental competence of hESC-derived endodermal lineage intermediates. *Cell Stem Cell.* 2015;16(4):386–99. 10.1016/j.stem.2015.02.013 25842977 PMC4478079

[164] Weedon MN Cebola I Patch AM: Recessive mutations in a distal *PTF1A* enhancer cause isolated pancreatic agenesis. *Nat Genet.* 2014;46(1):61–4. 10.1038/ng.2826 24212882 PMC4131753

[165] Sellick GS Barker KT Stolte-Dijkstra I: Mutations in *PTF1A* cause pancreatic and cerebellar agenesis. *Nat Genet.* 2004;36(12):1301–5. 10.1038/ng1475 15543146

[166] Schwitzgebel VM Mamin A Brun T: Agenesis of human pancreas due to decreased half-life of insulin promoter factor 1. *J Clin Endocrinol Metab.* 2003;88(9):4398–406. 10.1210/jc.2003-030046 12970316

[167] Stoffers DA Zinkin NT Stanojevic V: Pancreatic agenesis attributable to a single nucleotide deletion in the human *IPF1* gene coding sequence. *Nat Genet.* 1997;15(1):106–10. 10.1038/ng0197-106 8988180

[168] Burlison JS Long Q Fujitani Y: Pdx-1 and Ptf1a concurrently determine fate specification of pancreatic multipotent progenitor cells. *Dev Biol.* 2008;316(1):74–86. 10.1016/j.ydbio.2008.01.011 18294628 PMC2425677

[169] Dunne MJ Cosgrove KE Shepherd RM: Hyperinsulinism in infancy: from basic science to clinical disease. *Physiol Rev.* 2004;84(1):239–75. 10.1152/physrev.00022.2003 14715916

[170] Kapoor RR Flanagan SE Arya VB: Clinical and molecular characterisation of 300 patients with congenital hyperinsulinism. *Eur J Endocrinol.* 2013;168(4):557–64. 10.1530/EJE-12-0673 23345197 PMC3599069

[171] Snider KE Becker S Boyajian L: Genotype and phenotype correlations in 417 children with congenital hyperinsulinism. *J Clin Endocrinol Metab.* 2013;98(2):E355–63. 10.1210/jc.2012-2169 23275527 PMC3565119

[172] Salisbury RJ Han B Jennings RE: Altered Phenotype of β-Cells and Other Pancreatic Cell Lineages in Patients With Diffuse Congenital Hyperinsulinism in Infancy Caused by Mutations in the ATP-Sensitive K-Channel. *Diabetes.* 2015;64(9):3182–8. 10.2337/db14-1202 25931474 PMC4542438

[173] Collombat P Mansouri A Hecksher-Sorensen J: Opposing actions of Arx and Pax4 in endocrine pancreas development. *Genes Dev.* 2003;17(20):2591–603. 10.1101/gad.269003 14561778 PMC218152

[174] Papizan JB Singer RA Tschen SI: Nkx2.2 repressor complex regulates islet β-cell specification and prevents β-to-α-cell reprogramming. *Genes Dev.* 2011;25(21):2291–305. 10.1101/gad.173039.111 22056672 PMC3219233

[175] Rahier J Fält K Müntefering H: The basic structural lesion of persistent neonatal hypoglycaemia with hyperinsulinism: deficiency of pancreatic D cells or hyperactivity of B cells? *Diabetologia.* 1984;26(4):282–9. 10.1007/BF00283651 6376236

[176] Wills QF Boothe T Asadi A: Statistical approaches and software for clustering islet cell functional heterogeneity. *Islets.* 2016;8(2):48–56. 10.1080/19382014.2016.1150664 26909740 PMC4878268

